# Comparison of Internal Ribosome Entry Site (IRES) and Furin-2A (F2A) for Monoclonal Antibody Expression Level and Quality in CHO Cells

**DOI:** 10.1371/journal.pone.0063247

**Published:** 2013-05-21

**Authors:** Steven C. L. Ho, Muriel Bardor, Bin Li, Jia Juan Lee, Zhiwei Song, Yen Wah Tong, Lin-Tang Goh, Yuansheng Yang

**Affiliations:** 1 Bioprocessing Technology Institute, Agency for Science, Technology and Research, Singapore, Singapore; 2 Laboratoire Glyco-MEV (Glycobiologie et Matrice extracellulaire végétale), Université de Rouen, Mont-Saint-Aignan, France; 3 Department of Bioengineering, Department of Chemical and Biomolecular Engineering, National University of Singapore, Singapore, Singapore; 4 School of Chemical and Biomedical Engineering, Nanyang Technological University, Singapore, Singapore; St. Jude Children's Hospital, United States of America

## Abstract

Four versions of tricistronic vectors expressing IgG1 light chain (LC), IgG1 heavy chain (HC), and dihydrofolate reductase (DHFR) in one transcript were designed to compare internal ribosome entry site (IRES) and furin-2A (F2A) for their influence on monoclonal antibody (mAb) expression level and quality in CHO DG44 cells. LC and HC genes are arranged as either the first or the second cistron. When using mAb quantification methods based on the detection antibodies against HC Fc region, F2A-mediated tricistronic vectors appeared to express mAb at higher levels than the IRES-mediated tricistronic vectors in both transient and stable transfections. Further analysis revealed that more than 40% of products detected in stably transfected pools generated using the two F2A-mediated tricistronic vectors were aggregates. LC and HC from the F2A stably transfected pools were not properly processed, giving rise to LC+F2A+HC or HC+F2A+LC fusion proteins, LC and HC polypeptides with F2A remnants, and incorrectly cleaved signal peptides. Both IRES-mediated tricistronic vectors express mAb with correct sizes and signal peptide cleavage. Arrangement of LC as the first cistron in the IRES-mediated tricistronic vectors exhibits increased mAb expression level, better growth, and minimized product aggregation, while arrangement of HC as first cistron results in low expression, slower growth, and high aggregation. The results obtained will be beneficial for designing vectors that enhance mAb expression level and quality in mammalian cells.

## Introduction

Monoclonal antibodies (mAbs) are currently the fastest growing class of biotherapeutic molecules [1], [2]. Most mAbs in the market are immunoglobulin G (IgG) consisting of two identical heavy chain (HC) and two identical light chain (LC) polypeptides assembled via disulfide bridges. mAbs are commonly produced by stable transfection of Chinese hamster ovary (CHO) cells with the HC, LC and selection marker on either one or two separate vectors [3]–[6]. CHO DG44 cells are commonly used due to their compatibility with dihydrofolate reductase (DHFR), an amplifiable selection marker. Each gene is under the control of its own promoter and transcribed separately. One disadvantage of such designs is that vector fragmentation could result in non-expressing clones surviving drug selection [7], [8]. The other disadvantage is the lack of control over the ratio of LC:HC expression. LC is required to facilitate the folding and release of HC from BiP to form a complete IgG monomer [9]. It has been demonstrated that expression of LC in excess was beneficial for mAb expression [10]–[15]. The ratio of LC:HC expression can also affect mAb qualities such as aggregation and glycosylation [12], [15], [16]. Having HC in excess can cause ER stress [17] and proteasome overloading [18], creating a burden to the cell machinery and can inhibit cell proliferation [15].

Tricistronic vectors that express LC, HC, and selection marker genes in one mRNA are able to alleviate the above problems of traditional vectors. When vectors get fragmented, the mRNA unit would be incomplete and no genes would be expressed. Internal ribosome entry site (IRES) elements, which have a length of several hundred base pairs, allow expression of multiple genes in one mRNA. When IRES elements are included between multiple open reading frames (ORFs), the first ORF is translated by the canonical cap-dependent mechanism while the rest are translated through a cap-independent mechanism [19], [20]. The IRES-driven cap-independent translation has lower efficiency than the cap-dependent translation, resulting in lower expression of IRES-driven genes [21]–[23]. A few studies have used IRES for expressing mAb in mammalian cells [12], [24]–[27]. It has been demonstrated that an IRES-mediated tricistronic vector expressing LC, HC and neomycin in one transcript reduced the occurrence of non-expressing clones and controlled the LC:HC ratios at similar levels for all clones [12]. Clones generated using this vector expressed mAb at high levels with low aggregation and consistent glycosylation [12].

An alternative approach for co-expressing multiple genes in one mRNA is using 2A elements. 2A elements are much shorter than IRES, having only 60 to 80 base pairs. 2A linked genes are expressed in one single open reading frame and “self-cleavage” occurs co-translationally between the last two amino acids, GP, at the C-terminus of the 2A polypeptide, giving rise to equal amounts of co-expressed proteins [28]–[30]. The exact mechanism involved is still unclear but it has been suggested to involve a “ribosomal skip” between the two codons with no peptide bond formation between G and P [31]. Recent designs have added a furin cleavage sequence upstream of 2A to eliminate the additional amino acids which would otherwise remain attached to the upstream protein after cleavage [32], [33]. Furin-2A (F2A) elements have been used for mAb expression in mammalian cells [32]–[36] and for *in vivo* gene therapy [37]. It has been demonstrated that the productivities of F2A-vector derived clones were comparable with those derived from an industry reference vector based on separate expression unit design [36]. The design of the F2A vector used in that particular study was not released.

In studies conducted using F2A for mAb expression, mAb quality has only been characterized by western blotting. Detailed quality characterization of mAb expressed using F2A has not been reported. Only one study has compared IRES and F2A for mAb expression in transient transfections in HEK293 cells [32], in which the vector with a HC-F2A-LC arrangement gave higher mAb expression than the vector with a HC-IRES-LC arrangement. It is unclear which element is better when the positions of HC and LC are reversed. In this work, we sought to identify whether IRES or F2A works better for expression of humanized IgG1 in gene amplifiable CHO DG44 cells. We designed four tricistronic vectors expressing the LC, HC, and dihydrofolate reductase (DHFR) in one mRNA to compare transient and stable mAb expression in CHO DG44 cells. The quality of expressed mAb, including LC and HC polypeptide size, signal peptide cleavage, and aggregation, was characterized by western blotting, LC-MS/MS, and size exclusion chromatography (SEC), respectively.

## Materials and Methods

### Cell Culture and Media

Parental DHFR-deficient CHO DG44 cells (Life Technologies, Carlsbad, CA) were cultured in suspension using a protein-free medium consisting of HyQ PF (Hyclone, Logan, UT) and CD CHO (Life Technologies) at a 1∶1 ratio and supplemented with 2 g/L sodium bicarbonate (Sigma-Aldrich, St. Louis, MO), 6 mM glutamine (Sigma-Aldrich), 0.1% Pluronic F-68 (Life Technologies), and 1% hypoxanthine and thymine (HT) (Life Technologies). Regular passaging was carried out every 3 to 4 days in 125 mL shake flasks (Corning, NY) by diluting cells to 2×10^5^ cells/mL in 25 mL fresh medium. Cell density and viability were measured using the trypan blue exclusion method on an automated Cedex counter (Innovatis, Bielefeld, Germany).

### Vector Construction

**Figure 1:**
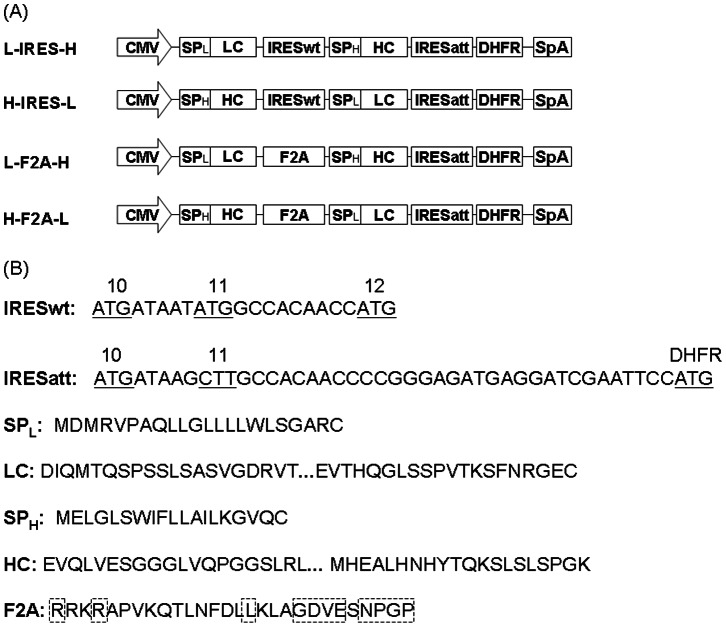
Schematic representation of the four tricistronic vectors for mAb expression. (A) Structure of different vectors. (B) DNA sequences of the 3′ end of IRESwt and IRESatt, amino acid sequences of signal peptide, F2A peptide, N- and C-termini of LC and HC are listed. The conserved regions of furin cleavage site and 2A peptide are highlighted in dotted boxes. CMV, human cytomegalovirus IE gene promoter; SpA, simian virus 40 early polyadenylation signal; IRESwt, wild type encephalomyocarditis virus (EMCV) internal ribosomal entry site; IRESatt, mutated EMCV IRES with attenuated translation efficiency; F2A, furin-2A peptide; LC, light chain cDNA; SP_L_, LC signal peptide; HC, heavy chain cDNA; SP_H_, HC signal peptide; DHFR, dihydrofolate reductase cDNA.

Two tricistronic vectors, L-IRES-H and H-IRES-L (Fig. 1), were obtained by replacing neomycin with DHFR in the previously described IRES-mediated tricistronic vectors expressing a biosimilar IgG1 mAb, Herceptin (anti-HER2) [12]. The IRES used to link the LC and HC genes is a wild-type encephalomyocarditis virus (EMCV) IRES (IRESwt). DHFR cDNA was cloned from the pSV2-DHFR vector (ATCC, Manassas, VA). Another two tricistronic vectors, L-F2A-H and H-F2A-L, were constructed by replacing the region of LC-IRESwt-HC with either LC-F2A-HC or HC-F2A-LC (Fig. 1). F2A, the furin cleavage sequence linked to the foot-and-mouth disease virus (FMDV) 2A sequence, was designed based on the literature [33]. The cDNA encoding F2A was synthesized by 1ST BASE (Singapore). The LC cDNA, F2A, and HC cDNA were assembled by overlapping PCR. F2A vectors expressing biosimilar IgG1 Humira (anti-TNFα) and Avastin (anti-VEGF) were generated by changing the LC and HC variable regions of the anti-HER2 vectors. The anti-TNFα and anti-VEGF variable region DNA were synthesized by Genescript (Piscataway, NJ). All restriction enzymes used were from New England Biolabs (Ipswich, MA).

### Transient Transfections

Transient transfection of DG44 cells were carried out using Nucleofector kit V and program U-24 on a Nucleofector I system (Lonza, Cologne, Germany). In each transfection, 2×10^6^ cells were co-transfected with 2 µg of the appropriate mAb encoding plasmid and 0.2 µg of a green fluorescence protein (GFP) encoding plasmid pMax-GFP (Lonza). The GFP plasmid acted as an internal control to account for variations in transfection efficiency. The transfected cells were then resuspended with 2 mL of HT-containing protein-free medium in 6-well suspension culture plates (Greiner Bio-One GmbH, Germany). Transfections were carried out in duplicates. At 48 h post-transfection, cells and supernatant were collected to measure GFP fluorescence intensity using a FACS Calibur (Becton Dickinson, MA) and to measure the mAb concentration using an enzyme-linked immunosorbent assay (ELISA) respectively. ELISA was performed as previously described using capture antibody of affinity purified goat anti-human IgA+IgG+IgM (HC+LC) (KPL, Gaithersburg, MD) and detection antibody of goat anti-human IgG (Fc specific) conjugated to alkaline phosphatase (Sigma-Aldrich) [10].

### Stable Transfections

Transfections to generate stably transfected pools were also performed using Nucleofector Kit V and program U-24 on a Nucleofector I system (Lonza). 1×10^7^ CHO DG44 cells were transfected with 5 µg of BglII linearized mAb expressing plasmid. Transfection for each of the four vectors was carried out in duplicates. The transfected cells were each incubated in 2 mL HT-containing protein-free medium in a 6-well suspension culture plate for 24 h. Samples were next centrifuged at ∼100×g for 5 min and cell pellets resuspended in 15 mL HT- and protein-free medium in 125 mL shake flasks to select for stable transfectants. Selection required around 2 to 3 weeks. When the stably transfected pools recovered with viability above 95%, gene amplification was induced by passaging in medium containing 50 nM methotrexate (MTX) (Sigma-Aldrich). The amplification process also required 2 to 3 weeks. Productivity of stable transfection pools at 50 nM of MTX was determined in 125 mL shake flask batch cultures. Cells were seeded in 25 mL of medium at a cell density of 2×10^5^ cells/mL. Growth was monitored every day until the end of culture when viability measurements dropped below 50%. Supernatant was collected at the end of culture and analyzed for mAb concentration using a nephelometric method on an IMMAGE 800 immunochemistry system (Beckman Coulter, Buckinghamshire, England). The IMMAGE 800 system utilized anti-human Fc region antibodies for IgG detection. The specific productivity (qmAb) was calculated as the mAb concentration divided by the integrated viable cell density (IVCD) which was determined based on the trapezoidal method.

### Western Blotting Analysis

Supernatants, which were collected from stably transfected pools at the end of culture, containing 10 ng of mAb as determined using the nephelometric method were mixed with NuPAGE sample loading buffer and reducing buffer (Life Technologies) and heated at 70°C for 2 min. Samples were loaded onto NuPAGE 4–12% Bis-Tris gel (Life Technologies) in MES buffer (Life Technologies). Precision plus protein dual color standards (Bio-Rad Laboratories, Hercules, CA) were used as molecular weight ladder and to check for transfer to the membrane. Proteins were transferred to polyvinylidene difluoride (PVDF) membranes (Life Technologies) using the iBlot system (Life Technologies). Membranes were blocked in 5% blocking milk (Bio-Rad Laboratories) in TBS with 0.1% Tween-20 (Bio-Rad Laboratories) for 1 h at room temperature and incubated overnight in HRP conjugated goat anti-human IgG Fc antibody (1∶5000; Bethyl Laboratories, Montgomery, TX) and HRP conjugated goat anti-human IgG Kappa LC (1∶20000; Bethyl Laboratories). Detection was done using ECL Prime (Amersham-GE Healthcare Life Sciences, Piscataway, NJ) and exposed on Lumi-Film Chemiluminescent Detection Film (Roche).

### Purification of mAb on Protein A Column

mAb in the supernatant collected at the end of culture of stable transfection pools was purified by affinity chromatography using protein A column on a GE AKTA explorer 100 (GE Healthcare, Uppsala, Sweden) as previously described [12]. This was carried out for duplicate transfected pools generated using each of the four tricistronic vectors.

### SDS-PAGE Separation of Protein A Purified Sample

Protein A purified mAb in stable transfection pools generated using different tricistronic vectors were separated by SDS-PAGE. Prior to SDS-PAGE separation, 10 µg of each purified mAb sample was denatured by boiling in the presence of 2-mercaptoethanol (Sigma-Aldrich) at 95°C for 5 min in the loading buffer containing 0.063 M Tris-HCl at pH of 6.8, 10.5% glycerol (BDH Chemicals, London, England), and 10% (w/v) SDS (Bio-Rad, Hercules, CA) and 0.1% bromophenol blue (GE Healthcare), and then ran at 15 mA for 90 min in a 10% T polyacrylamide gel (in-house created). Upon completion, the gel was immediately fixed and stained in coomassie blue composed of 0.3% (w/v) Coomassie Brilliant Blue R-250 (Peirce, St. Louis, IL) in 45% methanol and 10% acetic acid, and then washed with 10% methanol and 5% acetic acid to remove the background. The gel was scanned on Imagescanner III (GE Healthcare) and the relative intensities of the protein bands for each lane were quantified using the ImageQuant TL 7.0 software (GE Healthcare) based on the peak heights of each generated line graph.

### LC-MS/MS Analysis of Protein A Purified mAb

LC-MS/MS analysis was performed on each visible protein bands observed on the coomasie blue stained SDS-PAGE page gel. The bands on the gel were excised and washed in 150 µL of 50% acetonitrile (Fisher Scientific, Pittsburgh, PA) solution containing 25 mM ammonium bicarbonate. The washing solution was removed and the excised gels were dried using the Savant Speedvac (Savant Instruments, Holbrook, NY). Proteins within the excised gels were reduced by adding 25 µL of 20 mM DTT and incubated at 55°C for 60 min. Upon removal of the reducing agent, alkylation of the reduced proteins was facilitated by adding 25 µL of 55 mM iodoacetamide (Fluka, Steinheim, Germany) and incubated at room temperature in the dark for 45 min. The excised gels were then washed repeatedly in 100 mM ammonium bicarbonate solution, followed by 100% acetonitrile. After drying the excised gels using the Savant Speedvac, 20 µL of trypsin (Promega, Madison, WI) at 0.04 µg/µL in 25 mM ammonium bicarbonate solution was added to each excised gel sample. The enzyme-sample mixtures were incubated with agitation at 37°C overnight. Each digested sample was extracted by adding 20 µL of acetonitrile with sonication. Each acetonitrile solution containing digested proteins was transferred to a new clean vial and dried using the Savant Speedvac. Prior to injecting the samples onto the LC-MS/MS system, each sample was reconstituted in 5 µL of aqueous buffer containing 0.1% formic acid (Sigma-Aldrich) and 1% methanol (Fisher Scientific).

LC-MS/MS analyses were performed on a Velos Orbitrap mass spectrometer (Thermo Fisher Scientific, San Jose, CA) coupled to a nanoAcquity UPLC system (Waters, Milford, MA) fitted with a 180 µm × 20 mm Symmetry C18 peptide trap (Waters) and a 75 µm × 200 mm BEH130 C18 1.7-µm particle size analytical column (Waters). Depending on the peptide concentration of each sample, 1 to 2 µL of peptide digest was injected. Peptides were resolved by applying a linear binary gradient from 2 to 35% solvent B at 300 nL/min over 60 min at 35°C, where solvent A and B were 0.1% (v/v) formic acid in Milli-Q water (Millipore, Billerica, MA) and 0.1% formic acid in acetonitrile, respectively. To minimize sample carryover, a dedicated column wash run (10 to 95% B in 30 min) followed by column re-equilibration (2% B for 30 min) was performed prior to the next sample injection. The nano-ESI source was fitted with a 30-µm stainless steel nano-bore emitter (Thermo Fisher Scientific) with 1.7 kV applied near the tip. The MS instrument method used was the data-dependent acquisition mode that specified each orbitrap survey scan (at resolution 60,000) to be linked to a maximum of 10 MS/MS events; each with maximum ion trap fill time of 25 ms and isolation window of 2 m/z. The threshold for triggering an MS/MS was set at 500 counts. The ion trap CID fragmentation employed an activation time of 10 ms, q value of 0.25 and normalized collision energy of 35%. Charge state screening was enabled, with unknown and singly charged states excluded. Dynamic exclusion was enabled with a list size of 500 and exclusion time of 60 s.

Acquired LC-MS/MS data were processed by the Proteome Discoverer 1.3 software (Thermo Fisher Scientific) using the Sequest search engine. The peptide and fragment ion mass tolerances used were ±5 ppm and ±0.5 Da, respectively. The specified search parameters were carbamidomethylation of cysteine as fixed modification, oxidation of methionine as dynamic modification and tryptic digestion with 2 missed cleavages. Depending on the tricistronic vector configuration used (Fig. 1), each sample LC-MS/MS data was searched against the relevant full protein sequence to determine the actual expressed protein sequence for each excised gel band, with its corresponding sequence coverage calculated.

### Aggregation Analysis of Protein A Purified mAb

The aggregation of protein A purified mAb collected at the end of culture of stable transfection pools was determined using size exclusion chromatography (SEC) coupled to a UV-visible detector and a dynamic light scattering detector. Analysis was carried out for duplicate pools generated using each tricistronic vector. The instrument setup consisted of a HPLC system (Shimadzu, Kyoto, Japan) as previously described [12]. Several detectors including Dawn 8 (light scattering), Optilab (refractive index), and QELS (dynamic light scattering) were connected in series following a UV-visible detector. All the three detectors were purchased from Wyatt Technology Corporation (CA, USA) and were operated by the ASTRA software. The Chromatography columns used were TSK Guard column SWXL, 6×40 mm and TSK gel G3000 SWXL, 7.8×300 mm (Tosoh Corporation, Tokyo, Japan). The hydrodynamic radius measured by the light scattering detector was used to calculate the molecular weight of the different compounds present under each peak. The different fractions were assigned respectively to aggregates, monomer and fragments based on their measured molecular weights. The small rightmost peaks are a result of components in the buffer which the protein A purified samples are eluted in.

## Results

### Design of IRES- and F2A-mediated Tricistronic Vectors

An anti-HER2 IgG1 biosimilar was first used as a model mAb to compare IRES and F2A for mAb expression level and quality. Four tricistronic vectors were designed to express the anti-HER2 LC, anti-HER2 HC, and DHFR under the control of one CMV promoter (Fig. 1). LC and HC were arranged in either the first or the second cistron. The two vectors in which LC and HC are linked by a wild type EMCV IRES (IRESwt) are designated as L-IRES-H and H-IRES-L, respectively. Vectors in which LC and HC are linked by F2A are designated as L-F2A-H and H-F2A-L, respectively. The DHFR selection marker downstream of an attenuated EMCV IRES (IRESatt) in the third cistron was used for all the vectors for fair comparison. Application of IRESatt on DHFR will reduce its expression and can enhance stringency of selection for high producers [38]. Tight coupling of product gene and selection marker in one mRNA can reduce occurrence of non-expressing clones [12], [38], [39].

### Comparison of IRES and F2A for mAb Expression Level

**Figure 2:**
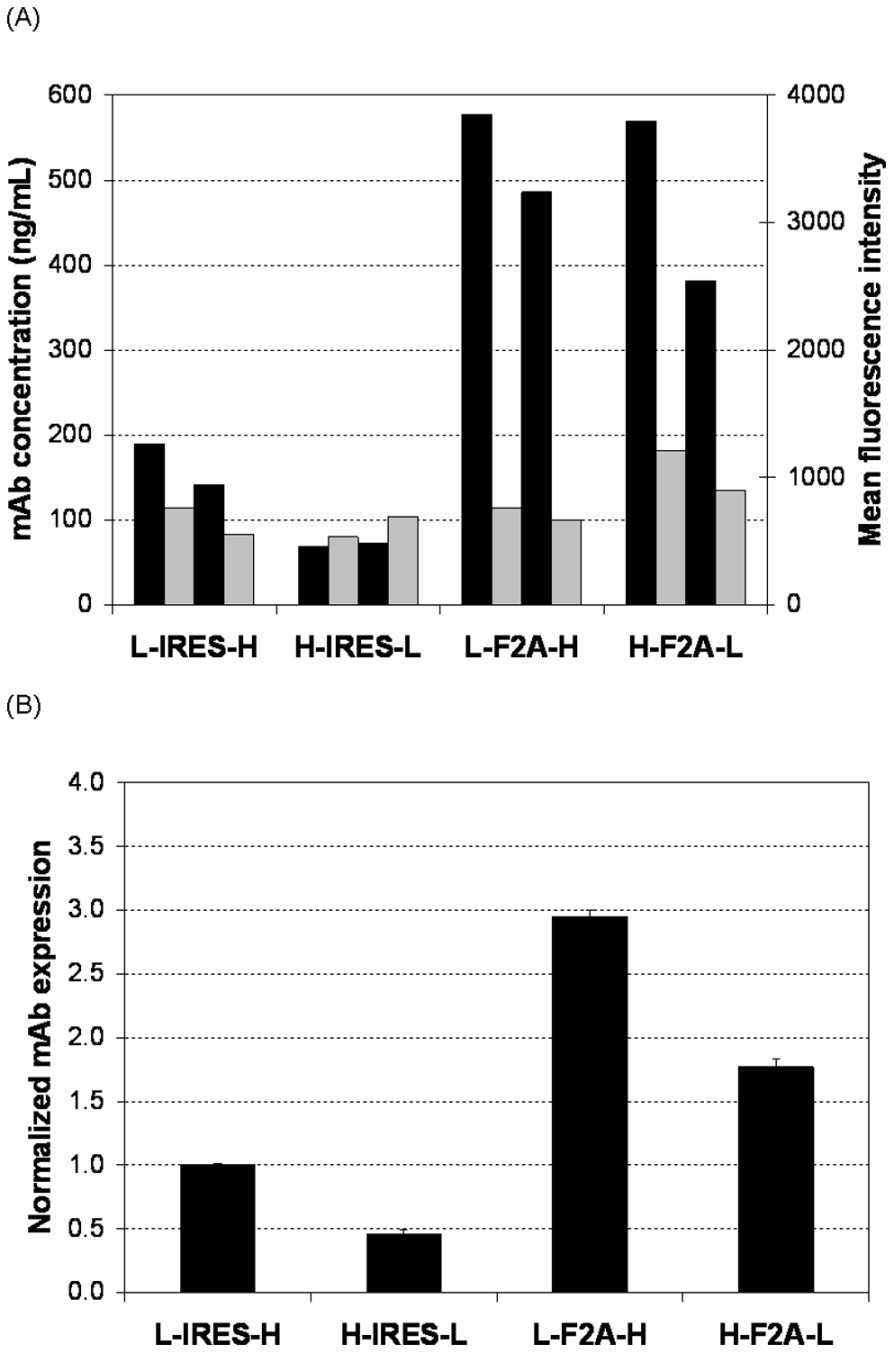
Comparison of the four tricistronic vectors for mAb expression in transient transfections. CHO DG44 cells were co-transfected with an appropriate mAb expressing vector containing IRES or F2A between the LC and HC gene and a GFP expressing vector. At 48 h post-transfection, supernatant and cells were collected for analysis of mAb concentration using ELISA and GFP expression using FACS, respectively. (A) Measured mAb concentration and GFP mean fluorescence intensity in each individual transfection. Black bars represent the mAb concentration and gray bars represent GFP mean fluorescence intensity. (B) Normalized mAb expression for each vector. Results represent mAb concentration measured in the culture supernatant normalized to the internal control, GFP expression to normalize the transfection efficiency and mAb expression from the L-IRES-H vector. Each point represents the average and standard deviation of two measurements from two independant transfections.

IRES and F2A were first compared for transient expression levels of anti-HER2 in 6-well plate cultures (Fig. 2A and 2B). As cap-dependent translation of the first cistron is more efficient than IRES-driven translation, the L-IRES-H vector will express LC in excess, and the H-IRES-L vector will express HC in excess. Consistent with previous reports that LC is more favorable for mAb expression [12], [15], [26], [40], we observed that the mAb expression level from the L-IRES-H vector to be around double that from the H-IRES-L vector. A previous study had reported that mAb expression from a F2A vector similar to H-F2A-L was greater than that from a EMCV IRES vector similar to H-IRES-L in transient transfections performed using HEK293 cells [32]. We observed that both F2A-mediated tricistronic vectors, showed higher mAb expression levels than those from the IRES-mediated tricistronic vectors regardless of LC and HC positions. L-F2A-H had the highest expression with titers at around 500 ng/mL, threefold higher than the titer of the better performing IRES vector, L-IRES-H.

**Figure 3:**
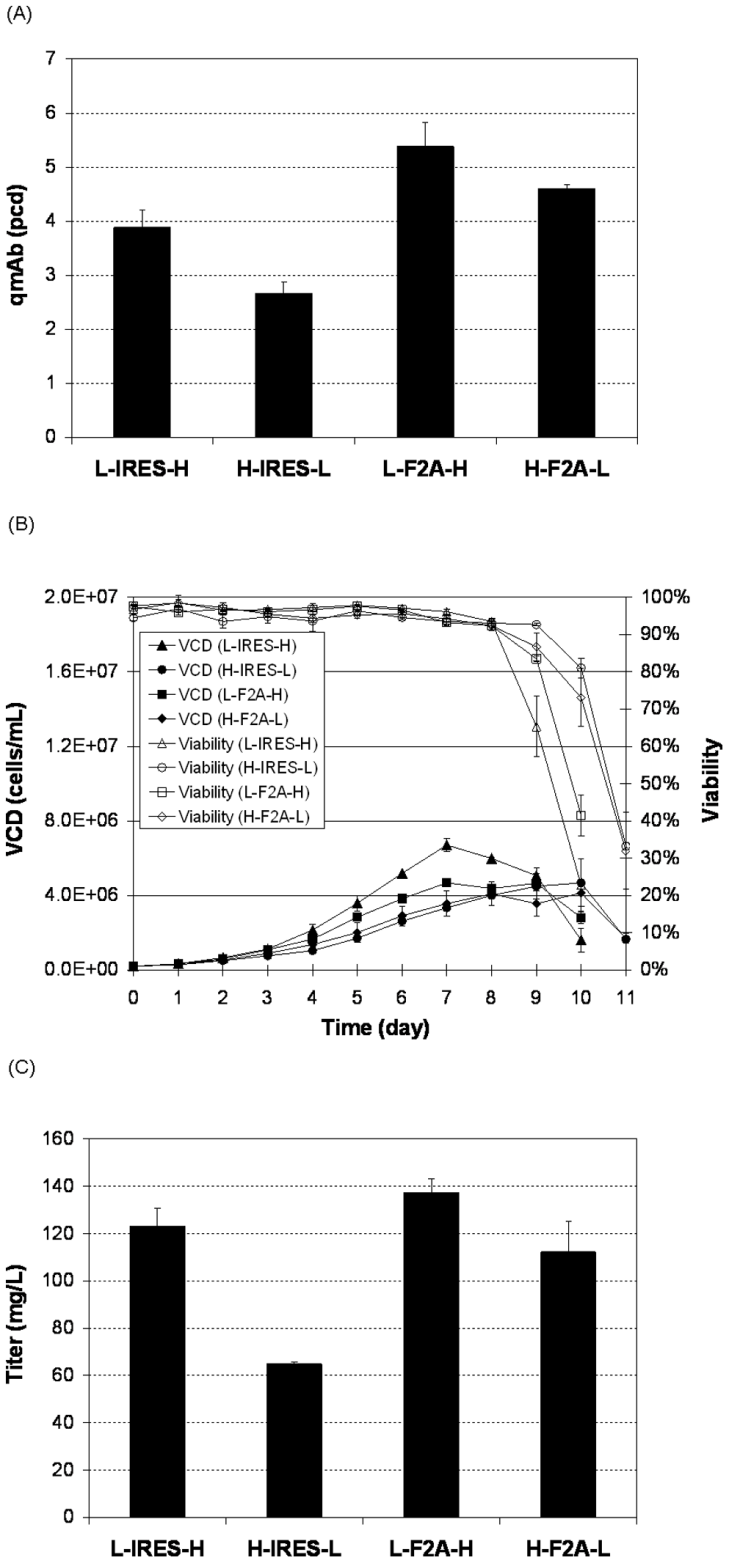
Comparison of the four tricistronic vectors for mAb expression in stable transfections. Stably transfected pools were generated by transfection of CHO DG44 cells with different tricistronic vectors containing either IRES or F2A between the LC and HC gene. Each pool was characterized for (A) specific mAb productivity (qmAb) measured in pg/cell/day (pcd), (B) growth data collected including viable cell density (VCD) and culture viability, and (C) titer. Each point represents the average and standard deviation of two measurements of two stably transfected pools.

Stable anti-HER2 expressions for the four vectors were next compared using shake flask batch cultures (Fig. 3A). Specific mAb productivity (qmAb) from the four vectors displayed a similar trend as that in transient transfections. The qmAb of the L-IRES-H stable pools was 3.9 pg/cell/day (pcd). The H-IRES-L stably transfected pools had lower qmAb of 2.7 pcd, which was 70% of the L-IRES-H stable pools. Both F2A-mediated tricistronic vectors still exhibited higher qmAb than the IRES-mediated tricistronic vectors. Compared to L-IRES-H, L-F2A-H qmAb was 1.4-fold and H-F2A-L was 1.2-fold higher, reaching 5.4 pcd and 4.4 pcd, respectively.

Stably transfected pools generated using the four vectors also exhibited differences in growth (Fig. 3B). The L-IRES-H stable pools grew fastest, followed by pools generated using the L-F2A-H, H-F2A-L and H-IRES-L vectors. The viability of the former two pools dropped below 50% at day 10, one day earlier than the latter two pools. The L-IRES-H stable pools also had highest peak cell density and IVCD, followed by L-F2A-H, H-F2A-L, and H-IRES-L. Due to the lower qmAb and even lower IVCD as compared to L-IRES-H, the H-IRES-L stable pool had 50% lower titer (Fig. 3C). The lower IVCD of the F2A pools also resulted in titers of L-F2A-H dropping to 1.1-fold higher than L-IRES-H, and H-F2A-L dropping lower than L-IRES-H.

### Western Blotting Analysis of mAb Products Expressed by IRES and F2A

**Figure 4:**
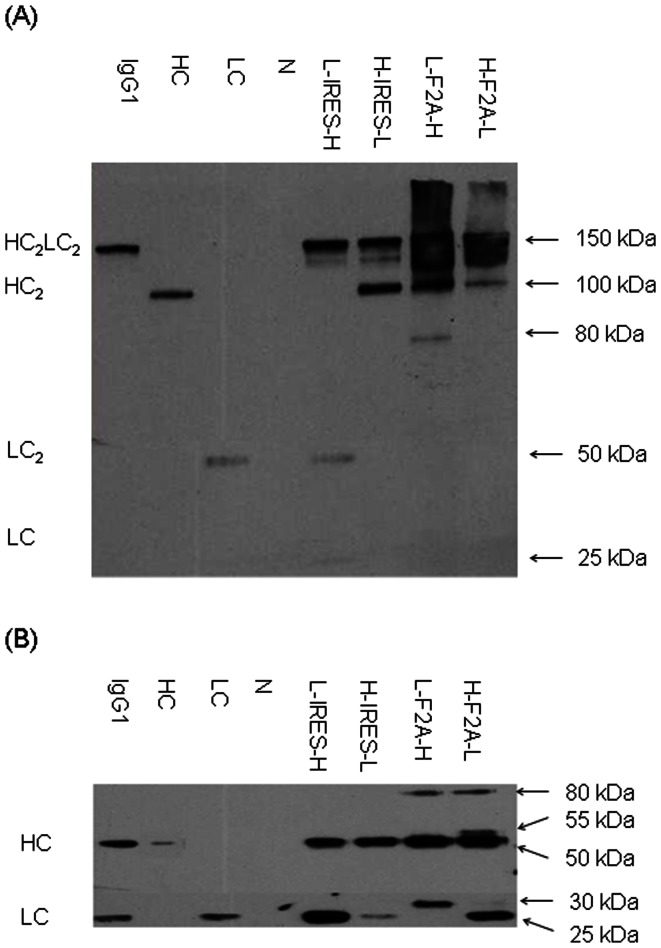
Western blot analysis of supernatant in stably transfected pools generated using the four tricistronic vectors. Each sample of crude supernatant was analyzed under both (A) non-reducing and (B) reducing conditions by western blot. A commercial human affinity purified myeloma Ig1 (Sigma-Aldrich) and supernatants from cells transfected with either a vector expressing only HC or a vector expressing only LC were used as positive control, and supernatant from non-transfected cells as negative control (N). All blots shown are only from the first replicates generated as duplicates were observed to exhibit the same product patterns.

Supernatant collected at the end of culture of CHO DG44 stably transfected pools generated using each of the tricistronic vectors were first analyzed under non-reducing conditions to characterize the products being secreted (Fig. 4A). Product from the L-IRES-H vector contained complete IgG1 monomers HC_2_LC_2_, LC_2_ dimer, and LC monomer, a sign that the L-IRES-H vector expressed LC in excess. Product from the H-IRES-L vector consisted of HC_2_LC_2_ complete IgG1 and HC_2_ dimers, providing evidence of expression of excess HC. Products from both F2A-mediated tricistronic vectors presented a smear with molecular weight greater than 100 kDa. The fractions above 150 kDa could correspond to mAb aggregates, and those around 150 kDa could be degraded IgG1 monomers, suggesting that mAb expressed from F2A was not stable. The two thin bands expressed from the L-F2A-H and H-F2A-L vectors with molecular weights of 100 kDa could be HC_2_, and the one band from the L-F2A-H vector with a molecular weight of 80 kDa could be a fusion protein of LC+F2A+HC.

Western blotting analysis of reduced products in the supernatant was next performed to identify the different components in the IgG1 being produced using the various vectors (Fig. 4B). Two bands were detected within supernatants from the two IRES-mediated tricistronic vectors, L-IRES-H and H-IRES-L. Their molecular weights corresponded to the standard HC and LC, respectively. Comparing the relative intensities of these two bands indicated that the L-IRES-H vector expressed LC in excess and the H-IRES-L vector expressed HC in excess. Interestingly, three bands were detected in reduced products from the L-F2A-H vector. The top band presents a molecular weight of approximately 80 kDa, which is equal to the sum of the molecular weights of LC, F2A, and HC suggesting that it is a fusion protein of LC+F2A+HC. The middle band exhibited similar size to the standard HC. The bottom band had slightly greater size than the standard LC, indicating a possible failed removal of 2A polypeptide residue by furin cleavage. Four bands were detected in products from the H-F2A-L vector. The top two bands could be the fusion proteins of HC+F2A+LC and HC+F2A based on their molecular weights. The other two bands had similar sizes to the standard HC and LC, respectively.

### LC-MS/MS Analysis of mAb Products Expressed by IRES and F2A

IRESwt has three ATGs at the 3′ end, which are designated as ATG-10, ATG-11, and ATG-12 as described previously [41]. ATG-12 is used as the start codon of mAb gene for higher translation efficiency [42]–[46]. Translation initiation of EMCV IRES-driven gene primarily occurs at ATG-11 and partially at ATG-12 [43]. Four extra amino acid residues, MATT, will be attached to the signal peptide of the IRES-driven LC or HC in those polypeptides which translation initiation occurs at ATG-11. “Self-cleavage” of 2A occurs between the last two amino acids, GP. Following self-cleavage, P will be attached to the signal peptide of LC or HC downstream of 2A and the rest of the 2A residues will attach to the C-terminus of the upstream gene. Addition of a furin cleavage site upstream of 2A can eliminate the 23 amino acid residues which would otherwise be attached to the HC or LC [32]. When HC is arranged upstream of F2A, design of a furin cleavage sequence with a second basic amino acid, such as RRKR, allowed carboxypeptidases to cleave both the RR residues left after furin cleavage together with the K on the C-terminus of HC [33].

**Figure 5:**
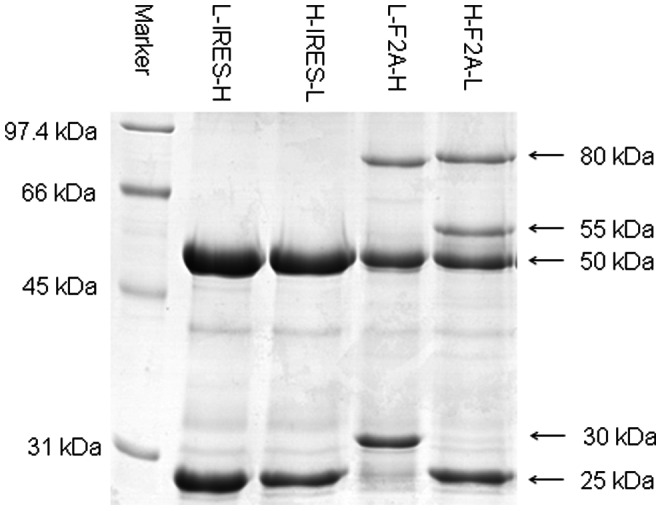
SDS-PAGE analysis of purified mAb in stably transfected pools generated using the four tricistronic vectors. Protein A purified supernatant was reduced and analyzed on a SDS-PAGE gel. Protein bands were visualized by coomasie blue staining. All gels shown are only from the first replicates generated as all duplicates were observed to exhibit the same product patterns.

To characterize the protein more accurately than just using western blotting, the reduced protein A purified mAb samples from duplicate pools of each vector were separated on SDS-PAGE (Fig. 5) and the bands were excised for LC-MS/MS analysis. The estimated molecular weight of each band, the polypeptide sequence as determined by peptide mapping and its associated sequence coverage, and the relative abundance of the expressed mAb chains are shown in Table 1. The various mAb chains were determined at high sequence coverage (>65%) with each N- and C-terminal sequence adequately detected (Figure S1).

**Table 1:** Relative abundance analysis of reduced antibody HC and LC variants by densitometry and sequence identity confirmation by peptide mapping.

Gel band	Peptide mapping detected polypeptides	L-IRES-H	L-IRES-H	H-IRES-L	H-IRES-L	L-F2A-H	L-F2A-H	H-F2A-L	H-F2A-L
(kDa)	(#Amino acids)	Sequencecoverage(%)	Relativeabundance(%)	Sequencecoverage(%)	Relativeabundance(%)	Sequencecoverage(%)	Relativeabundance(%)	Sequencecoverage(%)	Relativeabundance(%)
**25**	LC (214)	87.0±1.4	47.5±2.1	87.5±6.4	44.0±0.0	ND	ND	ND	ND
**25**	SP_L_+LC(236)	ND	ND	ND	ND	ND	ND	79.0±1.4	39.0±2.8
**30**	LC+F2A (242)	ND	ND	ND	ND	86.5±12.0	37.0±0.0	ND	ND
**50**	HC (450)	70.5±0.7	52.5±2.1	72.0±0.0	56.0±0.0	ND	ND	71.0±1.4	32.0±0.0
**50**	SP_H_+HC (469)	ND	ND	ND	ND	74.0±0.0	42.0±0.0	ND	ND
**55**	SP_H_+HC+F2A (496)	ND	ND	ND	ND	ND	ND	45.5±29.0	12.5±0.7
**80**	LC+F2A+SP_H_+HC (711)	ND	ND	ND	ND	67.0±2.8	21.0±0.0	ND	ND
**80**	SP_H_+HC+F2A+SP_L_+LC (733)	ND	ND	ND	ND	ND	ND	62.5±4.9	16.5±2.1

Each point represents the average and standard deviation of two measurements from two stable transfection pools. “ND” means not detected.

The excised gel bands from the L-IRES-H or H-IRES-L vector at molecular weights of 25 and 50 kDa were confirmed by LC-MS/MS analyses as LC and HC polypeptides, respectively. The excised gel bands from the L-F2A-H vector corresponding to molecular weights of 30, 50, and 80 kDa were LC and F2A fusion proteins (LC+F2A), HC with incorrect or uncleaved signal peptide (SP_H_+HC), and fusion proteins of LC+F2A+SP_H_+HC, respectively. The four excised gel bands from the H-F2A-L vector with molecular weights of 25, 50, 55 and 80 kDa were LC with incorrectly cleaved signal peptide (SP_L_+LC), HC, SP_H_+HC+F2A, and SP_H_+HC+F2A+SP_L_+LC, respectively. No incorrect signal peptide cleavage was observed on LC and HC in the IRES generated pools. The four extra amino acids which might attach to the signal peptides during translation initiation at IRES ATG-11 did not seem to affect the signal peptide cleavage. In contrast, incorrectly cleaved signal peptides were detected in both LC and HC expressed downstream of F2A as indicated by the detection of SP_L_+LC and SP_H_+HC with signal peptide residues attached. This is possibly due to attachment of the extra P from F2A to the signal peptide.

Cleavage of F2A was less efficient in products expressed from the L-F2A-H vector than the H-F2A-L vector. Occurrence of LC+F2A+HC or HC+F2A+LC fusion proteins indicates cleavage failure at both furin and 2A recognition sites. Existence of LC+F2A or HC+F2A indicates successful cleavage at the 2A recognition site but cleavage failure at the furin recognition site. 21% of the LC and HC expressed from the L-F2A-H vector existed as a LC+F2A+SP_H_+HC fusion protein. The rest were cleaved into similar abundances of LC+F2A (37%) and SP_H_+HC (42%) as expected. 16.5% of product expressed from the H-F2A-L vector was detected as SP_H_+HC+F2A+SP_L_+LC fusion proteins. The remaining was cleaved into products consisting of 32% HC, 12.5% SP_H_+HC+F2A, and 39% SP_L_+LC. The amount of SP_L_+LC was similar to the sum of HC and SP_H_+HC+F2A.

### Aggregation Analysis of mAb Products Expressed by IRES and F2A

**Figure 6:**
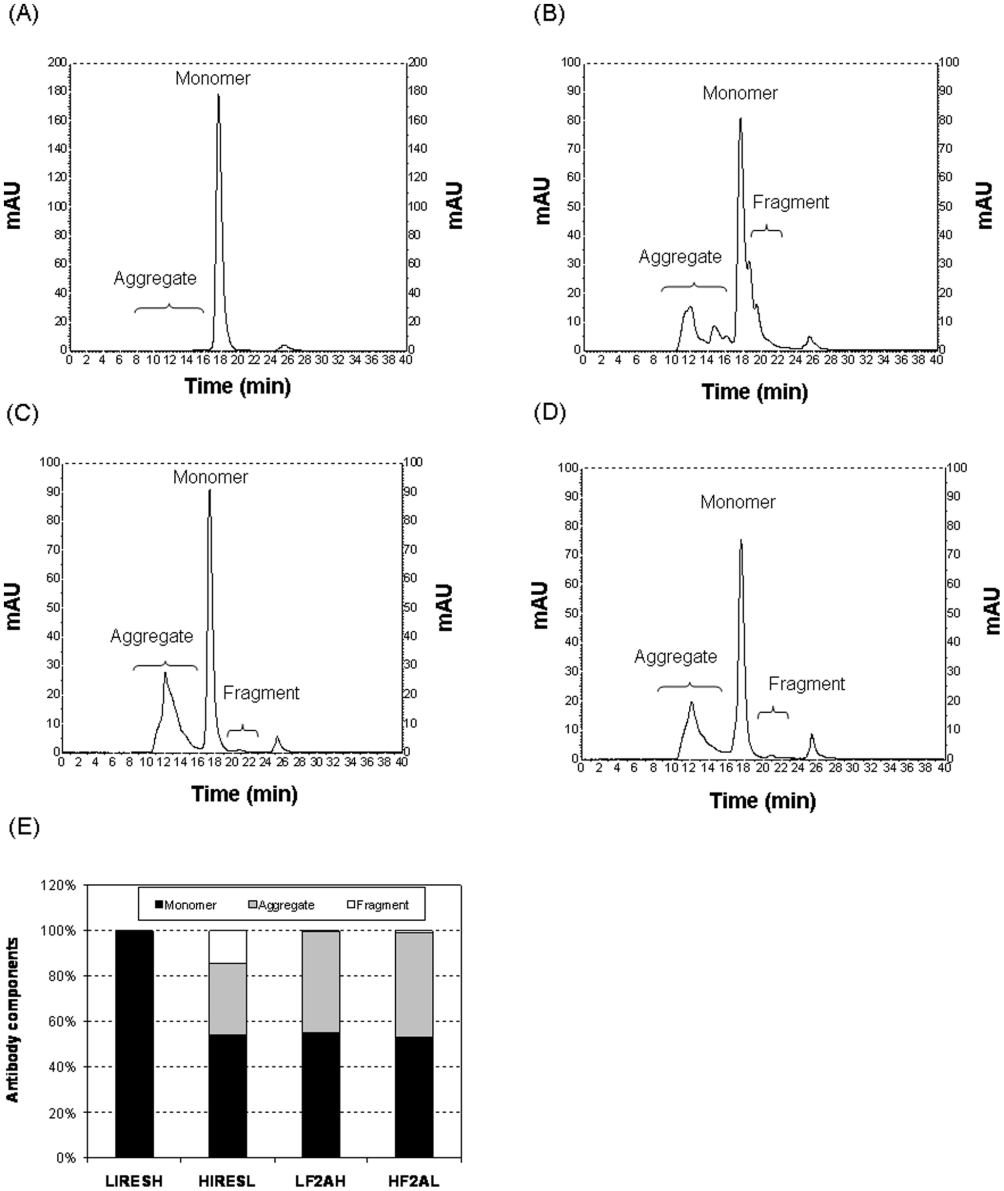
SEC analysis of protein A purified mAb in stably transfected pools generated using the four tricistronic vectors. Species within protein A purified mAb were separated by SEC followed by the identification and quantification of species by UV detection and light scattering, respectively. Analysis was done for duplicate stable transfection pools generated using each of the four tricistronic vectors. As results were consistent, only one typical chromatogram of the first pools analyzed from UV detector for vector (A) L-IRES-H, (B) H-IRES-L, (C) L-F2A-H, (D) H-F2A-L, are shown. (E) Quantitative comparison of aggregates, complete IgG1 monomers and incomplete IgG1 fragments of four vectors for different species. Each bar in figure E represents the average and standard deviation of four measurements from two stable transfection pools.

Aggregation of protein A purified anti-HER2 IgG1 mAb produced in stable transfection pools was analyzed and quantified using SEC coupled to a UV and a dynamic light scattering detector. As replicate pools gave identical chromatograms, only one representative UV chromatogram is shown for each vector (Fig. 6A to 6D). Molecular weight of each peak was calculated based on their respective hydrodynamic radius. Peaks with average molecular weight greater than the complete IgG1 monomer were grouped as aggregates and those with lower molecular weight were grouped as fragments. Relative mass amounts of aggregates, monomers and fragments were determined using the respective peak area under the UV chromatograms. The IgG1 monomer, aggregate and fragment distributions for each vector design is shown in figure 6E. The level of aggregates was less than 1% in products from the L-IRES-H vector. In contrast, products from H-IRES-L vector had 28% of aggregates and 13% of mAb fragments. Products from both F2A-mediated vectors had approximately 45% of aggregates and less than 1% of mAb fragments. In contrast to multiple aggregate peaks observed in H-IRES-L (Fig. 6B), F2A-mediated vectors only had one aggregate peak (Fig. 6C and 6D), suggesting different aggregation mechanisms. Interestingly, aggregates in H-IRES-L did not show up on western blots performed using the unpurified supernatant while aggregates in the F2A vectors were visible (Fig. 4A). This is possibly due to differences in the nature and components of the aggregates.

### Cleavage Efficiency of F2A for Other IgG1 mAbs

**Figure 7:**
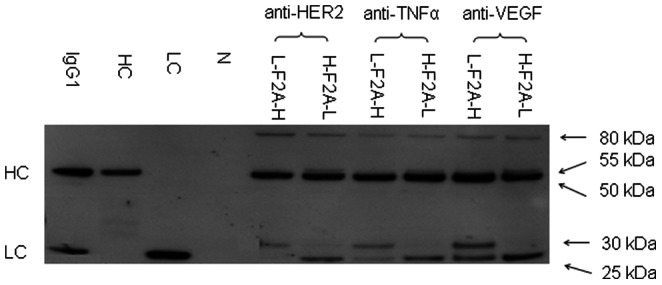
Western blot analysis of transiently expressed anti-HER2, anti-TNFα and anti-VEGF IgG1 mAbs. CHO DG44 cells were transfected with an appropriate mAb expressing tricistronic vector expressing anti-HER2, anti-TNFα, and anti-VEGF. At 48 h post-transfection, supernatant was collected for western blotting under reducing condition. The mAb loaded into each lane is identical as determined by ELISA. A commercial human affinity purified myeloma Ig1 (Sigma-Aldrich) and supernatants from cells transfected with either a vector expressing only HC or a vector expressing only LC were used as positive control, and supernatant from non-transfected cells as negative control (N). All blots shown are only from the first replicates generated as duplicates were observed to exhibit the same product patterns.

Previous reports which utilized F2A for expressing mAb did not report any issues with 2A peptide cleavage under their tested conditions [32]–[34], [36] while we observed inefficient F2A cleavage for anti-HER2 IgG1 expression in CHO DG44 cells. To investigate whether cleavage efficiency is mAb product dependent, we expressed two more IgG1 mAbs, anti-TNFα and anti-VEGF, in transient transfections and checked for cleavage efficiency using western blotting under reducing conditions (Fig. 7). Bands corresponding to LC+F2A+HC fusion protein, HC and LC+F2A fusion protein was observed for reduced anti-HER2 IgG1 expressed using the L-F2A-H vector and similar LC+F2A+HC, HC+F2A, HC and LC bands were observed for H-F2A-L. The products detected for anti-HER2 in the transient transfections are similar to that in products from stable transfections from both F2A vectors as shown earlier in figure 4B. Fusion proteins and uncleaved F2A residues were also observed for reduced samples of anti-TNFα and anti-VEGF. Reduced product bands similar to anti-HER2 were observed for the blots of both L-F2A-H and H-F2A-L except for an extra smaller band corresponding to LC for product from the L-F2A-H vectors in addition to the LC+F2A product band.

## Discussion

**Figure 8:**
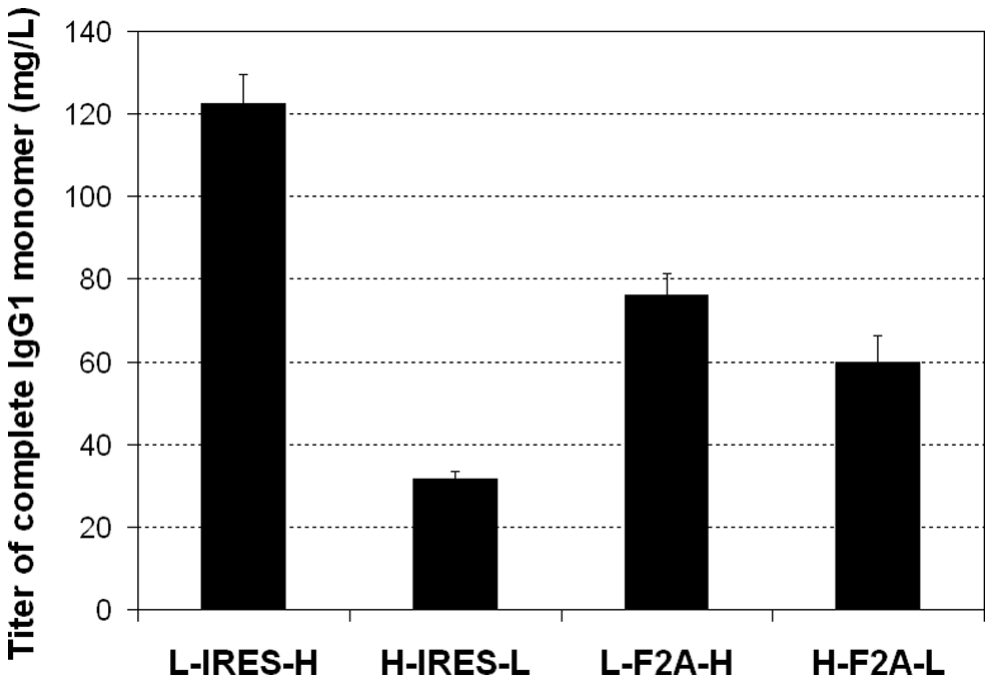
Estimation of the actual amount of complete IgG1 monomer produced in stably transfected pools generated using the four tricistronic vectors. The actual monomer amount was estimated based on the product titers measured by the nephelometer and the relative monomer peak area observed on SEC as shown in Figure 6E. Each point represent the average and standard deviation of two measurements from two stably transfected pools.

We designed four tricistronic vectors to compare the use of EMCV IRES and F2A for IgG1 mAb expression level and quality in CHO DG44 cells. The mAb quantification methods that we used in this work, ELISA and nephelometric methods are based on detection antibodies against the Fc region of the product mAb. Besides the complete IgG monomer, these assays would detect any components containing HC, such as high molecular weight aggregate and HC_2_ dimer, resulting in falsely high mAb titers. By measuring mAb in crude supernatant with these two assays, F2A vectors appeared to give higher mAb expression than IRES vectors in both transient and stable transfections. However, after considering the product distribution of complete IgG1 monomers, aggregates and fragments detected during SEC analysis (Fig. 6E), the amount of actual IgG1 monomer was significantly lower using H-IRES-L and the F2A constructs (Fig. 8). Actual titer in L-IRES-H stable transfection pools was unchanged and the highest at 123 mg/L, while titer of the H-IRES-L, L-F2A-H and H-F2A-L stable transfection pools dropped to 32 mg/L, 75 mg/L and 60 mg/L respectively.

Arrangement of LC as the first cistron gave higher mAb expression than arrangement of HC as the first cistron in both IRES- or F2A-mediated tricistronic vectors. This is understandable for the IRES-mediated tricistronic vectors as the L-IRES-H vector expressed LC in excess (Fig. 4) and extra LC is more favorable for mAb expression [12], [15], [26], [40]. Both L-F2A-H and H-F2A-L vectors expressed LC and HC in similar amounts (Table 1). One possible explanation is LC+F2A from the L-F2A-H vector can still be processed and does not create any burden to the cell’s protein folding and assembly machinery while HC+F2A from the H-F2A-L vector has detrimental effects. Another possible explanation is that LC+F2A+HC and HC+F2A+LC mRNAs have different secondary structures, and the former one has higher translation efficiency and thus yielding higher mAb expression level.

**Figure 9:**
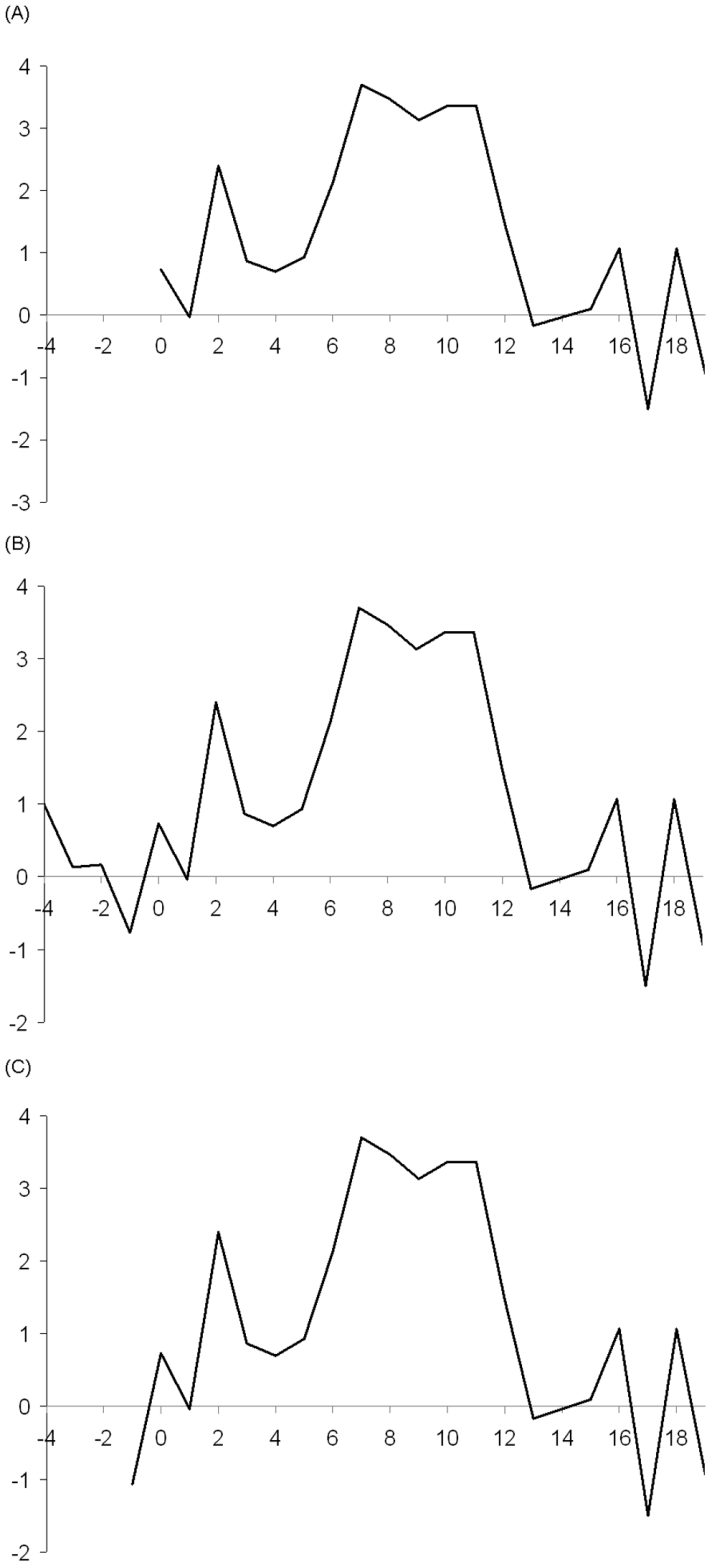
Hydrophobicity analysis of HC signal peptide attached with MATT and P amino acid residues at the N-terminal end. (A) Wild type HC signal peptide (SP_H_). (B) SP_H_ with MATT amino acid residues attached to the N-terminal end (MATT+SP_H_). (C) SP_H_ with amino acid P attached to the N-terminal end (P+SP_H_). The 19 amino acids of the HC signal peptide are shown as amino acids 0 to 19. Additional residues at the N-termini due to IRES and F2A processing are numbered starting from −1. Kyte-Doolittle index is computed using Protscale online tool (http://web.expasy.org/cgi-bin/protscale/protscale.pl).

Secretory proteins like the IgG1 LC and HC are bound by signal recognition particles (SRP) at their signal peptide sequence as they emerge from the ribosome within the cytosol and are targeted to SRP receptors of translocon complex on the ER membrane for translocation into the ER [47]. Each signal peptide sequence is separated into three regions: a hydrophilic and net-positively charged N-terminal region, a central hydrophobic region and a polar C-terminal region [48]. Studies have shown that an increase in the hydrophobicity of the N-terminal region can cause cleavage of the signal peptide to shift upstream [49]. In our vector designs, four additional amino acid residues, MATT, would be added to the IRES driven LC or HC peptides and one additional proline residue would be added for F2A driven peptides. Hydrophobicity index of the 19 amino acid HC signal peptide (SP_H_), HC signal peptide with MATT residues (MATT+SP_H_) and HC signal peptide with an additional P (P+SP_H_) were analyzed using the Kyte and Doolittle hydrophobicity index (Fig. 9) [50]. MATT residues would cause a lower net change in hydrophobicity of 0.533 as compared to addition of only one P residue causing a change of −1.067. This larger increased difference in hydrophobicity due to the P residue from F2A could be the reason causing incorrect cleavage of the signal peptide. Another reason for poor signal peptide cleavage could be due to the F2A reaction being inhibited by interactions between the nascent protein structure and the translocon complex [51]. SRP binding to the signal peptide downstream of F2A does not occur and in turn proper signal peptide cleavage is inhibited. It is also interesting to note that signal peptide cleavage prediction using SignalP 4.1 [52] did not indicate any changes to the cleavage site even with the additional residues.

Incomplete cleavage of F2A had been observed when used with various fluorescent reporter proteins [51], [53]. Previous reports of using F2A successfully for mAb expression had been in CHOK1 [33], [36], CHOK1SV [34] and HEK 293 [32] cells and none of the reports stated that they were using humanized IgG1 as model mAb. This is the first report of using the F2A for humanized IgG1 expression in CHO DG44 cells and the possibility of cell and product specificity affecting cleavage should also not be discounted. All three humanized IgG1 products expressed using the F2A system in CHO DG44 cells exhibited the presence of incompletely processed LC and HC products showing that the processing issues was not specific to only the anti-HER2 mAb that was initially characterized. While the 2A peptide cleavage was an issue regardless of product or arrangement, we made some interesting observations regarding the furin cleavage. Firstly, the different cistron arrangements affected the furin cleavage efficiency. Arrangement of LC upstream of F2A inhibited cleavage efficiency more than arrangement of HC upstream of F2A. This can be seen from the greater abundance of uncleaved LC+F2A in the L-F2A-H pools as compared to HC+F2A in the H-F2A-L pools in the western blot (Fig. 4B). Secondly, furin cleavage appeared to be dependent on 2A processing as we did not observe fusion proteins with F2A attached to the N-terminal of the protein translated from the second cistron, e.g. F2A+HC or F2A+LC. Thirdly, the furin cleavage efficiency was higher for anti-TNFα and anti-VEGF as compared to anti-HER2 in the L-F2A-H vectors. It is unclear why this was observed as the C-terminal ends of the LC attached to the furin cleavage sequence were the same for all three IgG1 products. One reason could be due to differences in structure of the complete IgG1 monomers due to different variable regions. IgGs are typically fully assembled as HC_2_LC_2_ in the ER [54] while furin endoproteases are mainly localized to the golgi [55]. The differences in structure of the three products within the golgi could affect furin cleavage efficiency. Less variation was observed for furin cleavage of the HC possibly due to the furin cleavage site being located further away from the differing Fab region.

The L-IRES-H was the best vector design for expressing IgG1 mAb in CHO DG44 cells among the four versions of tricistronic vectors. While the F2A system is a promising design, issues with 2A processing and furin cleavage affected product yield and quality. Further optimization of the type of 2A peptide to be used, addition of GSG linkers and the furin cleavage sequence need to be performed [33], [56]. It would be interesting for another comparison to be carried out after optimization.

## Supporting Information

Figure S1 **Peptide mapping of tryptic peptides of LC and HC expressed from the four tricistronic vectors.** Protein A purified product from duplicated stable transfection pools of each vector were analyzed. Purified samples from each pool were reduced and separated on SDS-PAGE. The bands (refer Figure 5) were excised for LC-MS/MS analysis. Sequences highlighted in green denote the tryptic peptides detected by LC-MS/MS. Underlined amino acid sequences are either signal peptide or F2A peptide. (A) Sample 1 and 2 produced from H-F2A-L vector corresponding to excised gel band at 80 kDa. (B) Sample 1 and 2 produced from H-F2A-L vector corresponding to excised gel band at 55 kDa band. (C) Sample 1 and 2 produced from H-F2A-L vector corresponding to excised gel band at 50 kDa band. (D) Sample 1 and 2 produced from H-F2A-L vector corresponding to excised gel band at 25 kDa band. (E) Sample 1 and 2 produced from L-F2A-H vector corresponding to excised gel band at 80 kDa band. (F) Sample 1 and 2 produced from L-F2A-H vector corresponding to excised gel band at 50 kDa band. (G) Sample 1 and 2 produced from L-F2A-H vector corresponding to excised gel band at 30 kDa band. (H) Sample 1 and 2 from H-IRES-L vector corresponding to excised gel band at 50 kDa band. (I) Sample 1 and 2 produced from H-IRES-L vector corresponding to excised gel band at 25 kDa band. (J) Sample 1 and 2 produced from L-IRES-H vector corresponding to excised gel band at 50 kDa band. (K) Sample 1 and 2 produced from L-IRES-H vector corresponding to excised gel band at 25 kDa band.
(DOC)
