# Supplementary material for: Comparison of Internal Ribosome Entry Site (IRES) and Furin-2A (F2A) for Monoclonal Antibody Expression Level and Quality in CHO Cells
Source: PLoS One. 2013 May 21;8(5):e63247. doi: 10.1371/journal.pone.0063247 (PMC3660568; doi:10.1371/journal.pone.0063247)
Supplement: Figure S1 — Peptide mapping of tryptic peptides of LC and HC expressed from the four tricistronic vectors. Protein A purified product from duplicated stable transfection pools of each vector were analyzed. Purified samples from each pool were reduced and separated on SDS-PAGE. The bands (refer Figure 5) were excised for LC-MS/MS analysis. Sequences highlighted in green denote the tryptic peptides detected by LC-MS/MS. Underlined amino acid sequences are either signal peptide or F2A peptide. (A) Sample 1 and 2 produced from H-F2A-L vector corresponding to excised gel band at 80 kDa. (B) Sample 1 and 2 produced from H-F2A-L vector corresponding to excised gel band at 55 kDa band. (C) Sample 1 and 2 produced from H-F2A-L vector corresponding to excised gel band at 50 kDa band. (D) Sample 1 and 2 produced from H-F2A-L vector corresponding to excised gel band at 25 kDa band. (E) Sample 1 and 2 produced from L-F2A-H vector corresponding to excised gel band at 80 kDa band. (F) Sample 1 and 2 produced from L-F2A-H vector corresponding to excised gel band at 50 kDa band. (G) Sample 1 and 2 produced from L-F2A-H vector corresponding to excised gel band at 30 kDa band. (H) Sample 1 and 2 from H-IRES-L vector corresponding to excised gel band at 50 kDa band. (I) Sample 1 and 2 produced from H-IRES-L vector corresponding to excised gel band at 25 kDa band. (J) Sample 1 and 2 produced from L-IRES-H vector corresponding to excised gel band at 50 kDa band. (K) Sample 1 and 2 produced from L-IRES-H vector corresponding to excised gel band at 25 kDa band. (DOC) [file pone.0063247.s001.doc]

**(A)**

MELGLSWIFL LAILK**GVQCE VQLVESGGGL VQPGGSLRLS CAASGFNIKD TYIHWVR**QAP GK**GLEWVARI YPTNGYTR**YA DSVKGR**FTIS ADTSKNTAYL**

**QMNSLRAEDT AVYYCSRWGG DGFYAMDYWG QGTLVTVSSA STKGPSVFPL APSSKSTSGG TAALGCLVK**D YFPEPVTVSW NSGALTSGVH TFPAVLQSSG

LYSLSSVVTV PSSSLGTQTY ICNVNHKPSN TKVDKKVEPK SCDK**THTCPP CPAPELLGGP SVFLFPPKPK** **DTLMISRTPE VTCVVVDVSH EDPEVKFNWY**

**VDGVEVHNAK** TKPREEQYNS TYR**VVSVLTV LHQDWLNGK**E YKCKVSNKAL PAPIEKTISK AKGQPR**EPQV YTLPPSR**DEL TK**NQVSLTCL VK**GFYPSDIA

VEWESNGQPE NNYK**TTPPVL DSDGSFFLYS K**LTVDKSRWQ QGNVFSCSVM HEALHNHYTQ KSLSLSPGKR RKR**APVKQTL NFDLLKLAGD VESNPGPMDM**

R**VPAQLLGLL LLWLSGARCD IQMTQSPSSL SASVGDR**VTI TCR**ASQDVNT AVAWYQQKPG K**APK**LLIYSA SFLYSGVPSR** FSGSR**SGTDF TLTISSLQPE**

**DFATYYCQQH YTTPPTFGQG TK**VEIKR**TVA APSVFIFPPS DEQLKSGTAS VVCLLNNFYP R**EAKVQWK**VD NALQSGNSQE SVTEQDSKDS TYSLSSTLTL**

**SK**ADYEKHK**V YACEVTHQGL SSPVTK**SFNR GEC

MELGLSWIFL LAILK**GVQCE VQLVESGGGL VQPGGSLRLS CAASGFNIKD TYIHWVR**QAP GK**GLEWVARI YPTNGYTR**YA DSVKGR**FTIS ADTSKNTAYL**

**QMNSLRAEDT AVYYCSRWGG DGFYAMDYWG QGTLVTVSSA STKGPSVFPL APSSKSTSGG TAALGCLVK**D YFPEPVTVSW NSGALTSGVH TFPAVLQSSG

LYSLSSVVTV PSSSLGTQTY ICNVNHKPSN TKVDKKVEPK SCDKTHTCPP CPAPELLGGP SVFLFPPKPK **DTLMISRTPE VTCVVVDVSH EDPEVKFNWY**

**VDGVEVHNAK** TKPREEQYNS TYR**VVSVLTV LHQDWLNGK**E YKCKVSNK**AL PAPIEKTISK** AKGQPR**EPQV YTLPPSR**DEL TK**NQVSLTCL VKGFYPSDIA**

**VEWESNGQPE NNYKTTPPVL DSDGSFFLYS K**LTVDKSRWQ QGNVFSCSVM HEALHNHYTQ KSLSLSPGKR RKR**APVKQTL NFDLLKLAGD VESNPGPMDM**

RVPAQLLGLL LLWLSGAR**CD IQMTQSPSSL SASVGDR**VTI TCR**ASQDVNT AVAWYQQKPG K**APK**LLIYSA SFLYSGVPSR** FSGSRSGTDF TLTISSLQPE

DFATYYCQQH YTTPPTFGQG TKVEIKR**TVA APSVFIFPPS DEQLKSGTAS VVCLLNNFYP R**EAKVQWK**VD NALQSGNSQE SVTEQDSKDS TYSLSSTLTL**

**SK**ADYEKHK**V YACEVTHQGL SSPVTK**SFNR GEC

**(B)**

MELGLSWIFL LAILK**GVQCE VQLVESGGGL VQPGGSLRLS CAASGFNIKD TYIHWVRQAP GKGLEWVARI YPTNGYTR**YA DSVKGR**FTIS ADTSKNTAYL**

**QMNSLRAEDT AVYYCSRWGG DGFYAMDYWG QGTLVTVSSA STKGPSVFPL APSSKSTSGG TAALGCLVK**D YFPEPVTVSW NSGALTSGVH TFPAVLQSSG

LYSLSSVVTV PSSSLGTQTY ICNVNHKPSN TKVDKKVEPK SCDK**THTCPP CPAPELLGGP SVFLFPPKPK** **DTLMISRTPE VTCVVVDVSH EDPEVKFNWY**

**VDGVEVHNAK** TKPR**EEQYNS TYRVVSVLTV LHQDWLNGK**E YKCKVSNKAL PAPIEKTISK AKGQPR**EPQV YTLPPSR**DEL TK**NQVSLTCL VK**GFYPSDIA

VEWESNGQPE NNYK**TTPPVL DSDGSFFLYS KLTVDK**SRWQ QGNVFSCSVM HEALHNHYTQ K**SLSLSPGK**R RKR**APVKQTL NFDLLKLAGD VESNPGP**

MELGLSWIFL LAILKGVQCE VQLVESGGGL VQPGGSLR**LS CAASGFNIK**D TYIHWVRQAP GKGLEWVARI YPTNGYTRYA DSVKGRFTIS ADTSKNTAYL

QMNSLR**AEDT AVYYCSRWGG DGFYAMDYWG QGTLVTVSSA STKGPSVFPL APSSKSTSGG TAALGCLVK**D YFPEPVTVSW NSGALTSGVH TFPAVLQSSG

LYSLSSVVTV PSSSLGTQTY ICNVNHKPSN TKVDKKVEPK SCDKTHTCPP CPAPELLGGP SVFLFPPKPK DTLMISRTPE VTCVVVDVSH EDPEVK**FNWY**

**VDGVEVHNAK** TKPREEQYNS TYRVVSVLTV LHQDWLNGKE YKCKVSNKAL PAPIEKTISK AKGQPREPQV YTLPPSRDEL TK**NQVSLTCL VK**GFYPSDIA

VEWESNGQPE NNYK**TTPPVL DSDGSFFLYS K**LTVDKSRWQ QGNVFSCSVM HEALHNHYTQ KSLSLSPGKR RKRAPVKQTL NFDLLKLAGD VESNPGP

**(C)**

MELGLSWIFL LAILKGVQCE VQLVESGGGL VQPGGSLR**LS CAASGFNIKD TYIHWVRQAP GKGLEWVARI YPTNGYTR**YA DSVK**GRFTIS ADTSKNTAYL**

**QMNSLRAEDT AVYYCSRWGG DGFYAMDYWG QGTLVTVSSA STKGPSVFPL APSSKSTSGG TAALGCLVK**D YFPEPVTVSW NSGALTSGVH TFPAVLQSSG

LYSLSSVVTV PSSSLGTQTY ICNVNHKPSN TKVDKKVEPK SCDK**THTCPP CPAPELLGGP SVFLFPPKPK** **DTLMISRTPE VTCVVVDVSH EDPEVKFNWY**

**VDGVEVHNAK** TKPR**EEQYNS TYRVVSVLTV LHQDWLNGK**E YKCK**VSNKAL PAPIEKTISK** AK**GQPREPQV YTLPPSR**DEL TK**NQVSLTCL VKGFYPSDIA**

**VEWESNGQPE NNYKTTPPVL DSDGSFFLYS KLTVDK**SR**WQ QGNVFSCSVM HEALHNHYTQ K**SLSLSPGK

MELGLSWIFL LAILKGVQCE VQLVESGGGL VQPGGSLR**LS CAASGFNIKD TYIHWVRQAP GKGLEWVARI YPTNGYTR**YA DSVKGR**FTIS ADTSKNTAYL**

**QMNSLRAEDT AVYYCSRWGG DGFYAMDYWG QGTLVTVSSA STKGPSVFPL APSSKSTSGG TAALGCLVK**D YFPEPVTVSW NSGALTSGVH TFPAVLQSSG

LYSLSSVVTV PSSSLGTQTY ICNVNHKPSN TKVDKKVEPK SCDK**THTCPP CPAPELLGGP SVFLFPPKPK** **DTLMISRTPE VTCVVVDVSH EDPEVKFNWY**

**VDGVEVHNAK** TKPR**EEQYNS TYRVVSVLTV LHQDWLNGK**E YKCK**VSNKAL PAPIEKTISK** AK**GQPREPQV YTLPPSR**DEL TK**NQVSLTCL VKGFYPSDIA**

**VEWESNGQPE NNYKTTPPVL DSDGSFFLYS K**LTVDKSR**WQ QGNVFSCSVM HEALHNHYTQ K**SLSLSPGK

**(D)**

MDMRVPAQLL GLLLLWLSGA R**CDIQMTQSP SSLSASVGDR** VTITCR**ASQD VNTAVAWYQQ KPGK**APK**LLI YSASFLYSGV PSR**FSGSR**SG TDFTLTISSL**

**QPEDFATYYC QQHYTTPPTF GQGTK**VEIK**R TVAAPSVFIF PPSDEQLKSG TASVVCLLNN FYPR**EAK**VQW KVDNALQSGN SQESVTEQDS KDSTYSLSST**

**LTLSKADYEK** HK**VYACEVTH QGLSSPVTK**S FNRGEC

MDMRVPAQLL GLLLLWLSGA R**CDIQMTQSP SSLSASVGDR** VTITCR**ASQD VNTAVAWYQQ KPGKAPKLLI YSASFLYSGV PSR**FSGSR**SG TDFTLTISSL**

**QPEDFATYYC QQHYTTPPTF GQGTK**VEIK**R TVAAPSVFIF PPSDEQLKSG TASVVCLLNN FYPR**EAKVQW K**VDNALQSGN SQESVTEQDS KDSTYSLSST**

**LTLSKADYEK** HK**VYACEVTH QGLSSPVTKS FNR**GEC

**(E)**

MDMRVPAQLL GLLLLWLSGA RCDIQMTQSP SSLSASVGDR VTITCR**ASQD VNTAVAWYQQ KPGKAPKLLI YSASFLYSGV PSR**FSGSR**SG TDFTLTISSL**

**QPEDFATYYC QQHYTTPPTF GQGTK**VEIK**R TVAAPSVFIF PPSDEQLKSG TASVVCLLNN FYPR**EAKVQW K**VDNALQSGN SQESVTEQDS KDSTYSLSST**

**LTLSK**ADYEK HK**VYACEVTH QGLSSPVTK**S FNRGECRRKR **APVKQTLNFD LLK**LAGDVES NPGPMELGLS WIFLLAILK**G VQCEVQLVES GGGLVQPGGS**

**LRLSCAASGF NIKDTYIHWV R**QAPGK**GLEW VAR**IYPTNGY TRYADSVK**GR FTISADTSKN TAYLQMNSLR AEDTAVYYCS RWGGDGFYAM DYWGQGTLVT**

**VSSASTKGPS VFPLAPSSKS TSGGTAALGC LVK**DYFPEPV TVSWNSGALT SGVHTFPAVL QSSGLYSLSS VVTVPSSSLG TQTYICNVNH KPSNTKVDKK

VEPKSCDK**TH TCPPCPAPEL LGGPSVFLFP PKPKDTLMIS RTPEVTCVVV DVSHEDPEVK** **FNWYVDGVEV HNAK**TKPR**EE QYNSTYRVVS VLTVLHQDWL**

**NGKEYK**CKVS NKALPAPIEK TISKAKGQPR **EPQVYTLPPS RDELTKNQVS LTCLVKGFYP SDIAVEWESN GQPENNYKTT PPVLDSDGSF FLYSK**LTVDK

SR**WQQGNVFS CSVMHEALHN HYTQK**SLSLS PGK

MDMRVPAQLL GLLLLWLSGA RCDIQMTQSP SSLSASVGDR VTITCR**ASQD VNTAVAWYQQ KPGK**APK**LLI YSASFLYSGV PSR**FSGSR**SG TDFTLTISSL**

**QPEDFATYYC QQHYTTPPTF GQGTK**VEIK**R TVAAPSVFIF PPSDEQLKSG TASVVCLLNN FYPR**EAK**VQW KVDNALQSGN SQESVTEQDS KDSTYSLSST**

**LTLSK**ADYEK HK**VYACEVTH QGLSSPVTK**S FNRGECRRKR **APVKQTLNFD LLK**LAGDVES NPGPMELGLS WIFLLAILK**G VQCEVQLVES GGGLVQPGGS**

**LRLSCAASGF NIKDTYIHWV R**QAPGK**GLEW VAR**IYPTNGY TRYADSVK**GR FTISADTSKN TAYLQMNSLR AEDTAVYYCS RWGGDGFYAM DYWGQGTLVT**

**VSSASTKGPS VFPLAPSSKS TSGGTAALGC LVK**DYFPEPV TVSWNSGALT SGVHTFPAVL QSSGLYSLSS VVTVPSSSLG TQTYICNVNH KPSNTKVDKK

VEPKSCDK**TH TCPPCPAPEL LGGPSVFLFP PKPKDTLMIS RTPEVTCVVV DVSHEDPEVK** **FNWYVDGVEV HNAK**TKPR**EE QYNSTYRVVS VLTVLHQDWL**

**NGK**EYKCKVS NKALPAPIEK TISKAKGQPR **EPQVYTLPPS RDELTKNQVS LTCLVK**GFYP SDIAVEWESN GQPENNYK**TT PPVLDSDGSF FLYSK**LTVDK

SR**WQQGNVFS CSVMHEALHN HYTQK**SLSLS PGK

**(F)**

MELGLSWIFL LAILK**GVQCE VQLVESGGGL VQPGGSLRLS CAASGFNIKD TYIHWVRQAP GKGLEWVARI YPTNGYTR**YA DSVK**GRFTIS ADTSKNTAYL**

**QMNSLRAEDT AVYYCSRWGG DGFYAMDYWG QGTLVTVSSA STKGPSVFPL APSSKSTSGG TAALGCLVK**D YFPEPVTVSW NSGALTSGVH TFPAVLQSSG

LYSLSSVVTV PSSSLGTQTY ICNVNHKPSN TKVDKKVEPK SCDK**THTCPP CPAPELLGGP SVFLFPPKPK** **DTLMISRTPE VTCVVVDVSH EDPEVKFNWY**

**VDGVEVHNAK** TKPR**EEQYNS TYRVVSVLTV LHQDWLNGKE YK**CK**VSNKAL PAPIEK**TISK AK**GQPREPQV YTLPPSRDEL TKNQVSLTCL VKGFYPSDIA**

**VEWESNGQPE NNYKTTPPVL DSDGSFFLYS K**LTVDKSR**WQ QGNVFSCSVM HEALHNHYTQ K**SLSLSPGK

MELGLSWIFL LAILK**GVQCE VQLVESGGGL VQPGGSLRLS CAASGFNIKD TYIHWVRQAP GKGLEWVARI YPTNGYTR**YA DSVK**GRFTIS ADTSKNTAYL**

**QMNSLRAEDT AVYYCSRWGG DGFYAMDYWG QGTLVTVSSA STKGPSVFPL APSSKSTSGG TAALGCLVK**D YFPEPVTVSW NSGALTSGVH TFPAVLQSSG

LYSLSSVVTV PSSSLGTQTY ICNVNHKPSN TKVDKKVEPK SCDK**THTCPP CPAPELLGGP SVFLFPPKPK** **DTLMISRTPE VTCVVVDVSH EDPEVKFNWY**

**VDGVEVHNAK** TKPR**EEQYNS TYRVVSVLTV LHQDWLNGKE YK**CK**VSNKAL PAPIEK**TISK AK**GQPREPQV YTLPPSRDEL TKNQVSLTCL VKGFYPSDIA**

**VEWESNGQPE NNYKTTPPVL DSDGSFFLYS K**LTVDKSR**WQ QGNVFSCSVM HEALHNHYTQ K**SLSLSPGK

**(G)**

MDMRVPAQLL GLLLLWLSGA **RC**DIQMTQSP SSLSASVGDR **VTITCRASQD VNTAVAWYQQ KPGKAPKLLI YSASFLYSGV PSRFSGSRSG TDFTLTISSL**

**QPEDFATYYC QQHYTTPPTF GQGTK**VEIK**R TVAAPSVFIF PPSDEQLKSG TASVVCLLNN FYPR**EAKVQW K**VDNALQSGN SQESVTEQDS KDSTYSLSST**

**LTLSKADYEK** HK**VYACEVTH QGLSSPVTKS FNR**GECRRK**R APVKQTLNFD LLKLAGDVES NPGP**

MDMRVPAQLL GLLLLWLSGA RCDIQMTQSP SSLSASVGDR **VTITCRASQD VNTAVAWYQQ KPGKAPKLLI YSASFLYSGV PSRFSGSR**SG TDFTLTISSL

QPEDFATYYC QQHYTTPPTF GQGTKVEIK**R TVAAPSVFIF PPSDEQLKSG TASVVCLLNN FYPR**EAK**VQW KVDNALQSGN SQESVTEQDS KDSTYSLSST**

**LTLSKADYEK** HK**VYACEVTH QGLSSPVTK**S FNRGECRRKR **APVKQTLNFD LLKLAGDVES NPGP**

**(H)**

MELGLSWIFL LAILKGVQCE VQLVESGGGL VQPGGSLR**LS CAASGFNIKD TYIHWVRQAP GKGLEWVAR**I YPTNGYTRYA DSVK**GRFTIS ADTSKNTAYL**

**QMNSLRAEDT AVYYCSRWGG DGFYAMDYWG QGTLVTVSSA STKGPSVFPL APSSKSTSGG TAALGCLVK**D YFPEPVTVSW NSGALTSGVH TFPAVLQSSG

LYSLSSVVTV PSSSLGTQTY ICNVNHKPSN TKVDKKVEPK SCDK**THTCPP CPAPELLGGP SVFLFPPKPK** **DTLMISRTPE VTCVVVDVSH EDPEVKFNWY**

**VDGVEVHNAK TKPREEQYNS TYRVVSVLTV LHQDWLNGKE YK**CK**VSNKAL PAPIEK**TISK AK**GQPREPQV YTLPPSRDEL TKNQVSLTCL VKGFYPSDIA**

**VEWESNGQPE NNYKTTPPVL DSDGSFFLYS K**LTVDKSR**WQ QGNVFSCSVM HEALHNHYTQ K**SLSLSPGK

MELGLSWIFL LAILKGVQCE VQLVESGGGL VQPGGSLR**LS CAASGFNIKD TYIHWVR**QAP GK**GLEWVAR**I YPTNGYTRYA DSVK**GRFTIS ADTSKNTAYL**

**QMNSLRAEDT AVYYCSRWGG DGFYAMDYWG QGTLVTVSSA STKGPSVFPL APSSKSTSGG TAALGCLVK**D YFPEPVTVSW NSGALTSGVH TFPAVLQSSG

LYSLSSVVTV PSSSLGTQTY ICNVNHKPSN TKVDKKVEPK SCDK**THTCPP CPAPELLGGP SVFLFPPKPK** **DTLMISRTPE VTCVVVDVSH EDPEVKFNWY**

**VDGVEVHNAK TKPREEQYNS TYRVVSVLTV LHQDWLNGKE YK**CK**VSNKAL PAPIEK**TISK AK**GQPREPQV YTLPPSRDEL TKNQVSLTCL VKGFYPSDIA**

**VEWESNGQPE NNYKTTPPVL DSDGSFFLYS K**LTVDKSR**WQ QGNVFSCSVM HEALHNHYTQ K**SLSLSPGK

**(I)**

MDMRVPAQLL GLLLLWLSGA RCDIQMTQSP SSLSASVGDR **VTITCRASQD VNTAVAWYQQ KPGKAPKLLI YSASFLYSGV PSRFSGSRSG TDFTLTISSL**

**QPEDFATYYC QQHYTTPPTF GQGTK**VEIK**R TVAAPSVFIF PPSDEQLKSG TASVVCLLNN FYPR**EAK**VQW KVDNALQSGN SQESVTEQDS KDSTYSLSST**

**LTLSKADYEK** HK**VYACEVTH QGLSSPVTKS FNR**GEC

MDMRVPAQLL GLLLLWLSGA RCDIQMTQSP SSLSASVGDR **VTITCRASQD VNTAVAWYQQ KPGKAPKLLI YSASFLYSGV PSRFSGSRSG TDFTLTISSL**

**QPEDFATYYC QQHYTTPPTF GQGTK**VEIK**R TVAAPSVFIF PPSDEQLKSG TASVVCLLNN FYPREAKVQW KVDNALQSGN SQESVTEQDS KDSTYSLSST**

**LTLSKADYEK** **HKVYACEVTH QGLSSPVTKS FNR**GEC

**(J)**

MELGLSWIFL LAILKGVQCE VQLVESGGGL VQPGGSLR**LS CAASGFNIKD TYIHWVR**QAP GK**GLEWVARI YPTNGYTR**YA DSVK**GRFTIS ADTSKNTAYL**

**QMNSLRAEDT AVYYCSRWGG DGFYAMDYWG QGTLVTVSSA STKGPSVFPL APSSKSTSGG TAALGCLVK**D YFPEPVTVSW NSGALTSGVH TFPAVLQSSG

LYSLSSVVTV PSSSLGTQTY ICNVNHKPSN TKVDKKVEPK SCDK**THTCPP CPAPELLGGP SVFLFPPKPK** **DTLMISRTPE VTCVVVDVSH EDPEVKFNWY**

**VDGVEVHNAK TKPREEQYNS TYRVVSVLTV LHQDWLNGKE YK**CK**VSNKAL PAPIEK**TISK AK**GQPREPQV YTLPPSRDEL TKNQVSLTCL VKGFYPSDIA**

**VEWESNGQPE NNYKTTPPVL DSDGSFFLYS K**LTVDKSR**WQ QGNVFSCSVM HEALHNHYTQ K**SLSLSPGK

MELGLSWIFL LAILKGVQCE VQLVESGGGL VQPGGSLR**LS CAASGFNIKD TYIHWVR**QAP GK**GLEWVARI YPTNGYTR**YA DSVK**GRFTIS ADTSKNTAYL**

**QMNSLRAEDT AVYYCSRWGG DGFYAMDYWG QGTLVTVSSA STKGPSVFPL APSSKSTSGG TAALGCLVK**D YFPEPVTVSW NSGALTSGVH TFPAVLQSSG

LYSLSSVVTV PSSSLGTQTY ICNVNHKPSN TKVDKKVEPK SCDK**THTCPP CPAPELLGGP SVFLFPPKPK** **DTLMISRTPE VTCVVVDVSH EDPEVKFNWY**

**VDGVEVHNAK TKPREEQYNS TYRVVSVLTV LHQDWLNGKE YK**CK**VSNKAL PAPIEKTISK** AK**GQPREPQV YTLPPSRDEL TKNQVSLTCL VKGFYPSDIA**

**VEWESNGQPE NNYKTTPPVL DSDGSFFLYS K**LTVDKSR**WQ QGNVFSCSVM HEALHNHYTQ K**SLSLSPGK

**(K)**

MDMRVPAQLL GLLLLWLSGA RCDIQMTQSP SSLSASVGDR **VTITCRASQD VNTAVAWYQQ KPGKAPKLLI YSASFLYSGV PSRFSGSRSG TDFTLTISSL**

**QPEDFATYYC QQHYTTPPTF GQGTKVEIKR TVAAPSVFIF PPSDEQLKSG TASVVCLLNN FYPREAKVQW KVDNALQSGN SQESVTEQDS KDSTYSLSST**

**LTLSKADYEK** **HKVYACEVTH QGLSSPVTKS FNRGEC**

MDMRVPAQLL GLLLLWLSGA RCDIQMTQSP SSLSASVGDR **VTITCRASQD VNTAVAWYQQ KPGKAPKLLI YSASFLYSGV PSRFSGSRSG TDFTLTISSL**

**QPEDFATYYC QQHYTTPPTF GQGTK**VEIK**R TVAAPSVFIF PPSDEQLKSG TASVVCLLNN FYPR**EAK**VQW KVDNALQSGN SQESVTEQDS KDSTYSLSST**

**LTLSK**ADYEK **HKVYACEVTH QGLSSPVTK**S FNRGEC
